# Anatomical Changes in Malarial and Yellow Fever

**Published:** 1898-06

**Authors:** Bat Smith

**Affiliations:** Wharton, Texas


For the Texas Medical Journal.

### Anatomical Changes in Malarial and Yellow Fever.

BY BAT SMITH, A. M., M. D., WHARTON, TEXAS.

[Read at the meeting of the East Texas Medical Association, Beaumont, Texas.]

The efforts of bacteriologists to find and determine the character of the germ of yellow fever either in the blood or the tissues, having led to negative results, the writer has been induced to submit to your consideration the following notes made by him many years ago. In so doing I have solely considered those points which may be of use to the ordinary practitioner, who, in many instances does not possess the appliances necessary for close scientific investigation. As stated above, these notes were made many years ago, and may contain many errors; if so, I would be pleased to submit them to the criticism of the members of the Association, assuring you that any corrections you may make will be most kindly and thankfully received.

In comparing the anatomical changes in malarial and yellow fever, I have selected that form of malarial fever which most closely resembles yellow fever, viz: Hemorrhagic malarial fever.

### APPEARANCE OF THE BODY IN HEMORRHAGIC MALARIAL FEVER.

The exterior of the body is generally of a yellow color, as a rule, darker than in yellow fever. Sometimes the skin is of a bronze color. There is generally quite an extensive discoloration of the more dependent portions of the trunk and extremities, due to hypostasis. The gums usually present a deep lead color. The yellow color of the skin is due to bile. The conjunctiva is also discolored by bile. Rigor mortis strongly marked.

### APPEARANCE OF THE BODY IN YELLOW FEVER.

Not reduced, appears swollen and bloated. The coloc of the body is yellow, somewhat brighter than in hemorrhagic malarial fever. The dependent portions of the trunk and extremities are of a purplish mottled hue. The conjunctiva is also of a yellow color, and black vomit is often seen oozing from the corners of the mouth and nose. Gums of a dark red color. The yellow color in yellow fever is due to free hæmatine, and not to bile. In testing pieces of the skin for bile, I have always found it absent. Rigor mortis strongly marked.

THE LUNGS IN HEMORRHAGIC MALARIAL FEVER.

Evidences of congestion of the lungs are occasionally found, but, as a rule, they present an apparently normal condition.

THE LUNGS IN YELLOW FEVER.

With the exception of the congestion usually found in the dependent positions, they are otherwise normal.

The heart in hemorrhagic malarial fever is firm and natural, of a purplish red color, the sarcous elements of its muscular structure seem to be in a normal condition. I have occasionally found fatty degeneration of the heart in malarial fever, but in these cases, the lesion was caused by chronic poisoning by alcohol; the degeneration in these cases was not complete. I have also frequently found febrinous clots in the heart, extending in some cases far into the aorta. These clots I have never found in yellow fever.

The heart in yellow fever. The pericardium presents a congested appearance, the capillaries being full of blood, and the pericardial fluid of a deep yellow color. The heart itself is soft and flabby, due to fatty degeneration, the sarcous elements of Bowman having undergone a true retrograde metamorphosis, and become converted from albumen into fat. Upon submitting a section of the heart to the microscope, the peculiar shining appearance of fat globules will be noticed throughout the structure. Upon the treatment of the specimen with ether, the fat globules disappear, but the sarcous elements are not to be found.

The stomach in hemorrhagic malarial fever gives evidence of congestion; at times we also find a quantity of blackish or blackish-green fluid of an acid reaction, and which contains a large amount of bilious matter, but with a limited number of blood corpuscles. The entire mucous membrane is discolored by bile. Upon washing the stomach the mucous membrane presents a mottled appearance, reddish brown in color, indicative more of capillary stagnation than of inflammatory process.

The stomach in yellow fever shows an intensely congested condition of the mucous coat, it is softened and shows many abrasions. In many cases the whole mucous coat is involved; then again this eroded condition is found more or less at the cardiac and pyloric portions, the other parts being comparatively free from abrasions. In the stomach, as a rule, a large quantity of black vomit is found, consisting of blood corpuscles, broken down capillaries, and cells of mucous membrane, bile being absent. The reaction of this black vomit is alkaline, owing to the large quantity of ammonia which it contains, produced by the decomposition of urea. The presence of ammonia can readily be demonstrated, by dipping a glass rod into muriatic acid and holding it over the black vomit, when immediately fumes of chloride of ammonium will arise.

The intestines in malarial fever present practically similar changes to those found in the stomach. The mucous membrane is often of a purplish, mottled appearance. Brunner's glands are sometimes found enlarged, while Peyer's patches are free from morbid changes or inflammation. Bile is found in the intestinal canal in large quantities, being absent from the entire alimentary tract in yellow fever.

The intestines in yellow fever are generally much distended with gas; they are dark colored, with a strong alkaline reaction. In many cases the intestines are distended with blood, owing to hemorrhages from the mucous membranes, although no discharges may have taken place during life. The large intestines and the lower portion of the small intestines are not so much congested as the stomach and upper portion of the small intestines. Professor John Harrison reports a curious and very remarkable condition of the intestines in yellow fever. In his numerous post mortem examinations made in New Orleans in 1839, he found intussusception of the small intestines an exceedingly common occurrence. I have only met with this condition once.

The spleen in malarial fever is usually enlarged far beyond its normal size. The pulp is of a dark, muddy color, soft so as to be easily torn, and though it does not turn red, yet it assumes a slightly brighter color when cut into and exposed to light. There are evidences of a profuse exudation within the parenchyma of the organ, accompanied by a rich deposit of pigment, imparting to it its peculiar dark muddy color. The capsule is frequently subject to inflammatory action.

The spleen in yellow fever was found, at times, to be of more than normal size; in these cases, however, evidence of preexisting malaria was shown. In uncomplicated cases of yellow fever the size of the organ was natural, or it was but very slightly enlarged. Unlike in malarial fever, there seemed to be but little alteration or destruction of the red blood corpuscles, they were not crenated, as they are to a great extent in malarial fever, neither are their number diminished in proportion to the white corpuscles, as is always the case in malarial fever. They are (the red corpuscles), however, much paler than usual.

Pancreas.—With the exception of numerous fat globules found throughout the structure of the organ, no appreciable alteration, as far as I could detect, were to be found in the pancreas either in malarial or yellow fever.

The liver in malarial fever in enlarged, of a dark slate color, and when cut into the color is a deep bronze. Under the microscope, a large number of dark pigment particles are to be seen; in many of the hepatic cells a large number of pigment granules are deposited.

The liver in uncomplicated cases of yellow fever is of a golden yellow color, and but little blood is to be found. The color varies with the length of the attack. Cases which have died early in the attack are frequently engorged with blood. Again, we meet with a mottled liver, the yellow color predominating. These are the cases which have suffered from previous diseases, especially from malarial fever. This golden yellow color which we find uniformly throughout the liver in yellow fever, is due to fatty degeneration of the protoplasm of the organ, and also to a fatty infiltration, the metamorphosis not always being complete. With some specimens of the liver placed under the microscope, I have noticed that the structure of the liver seemed entirely gone, yet, after washing the specimen with ether, the original structure would reappear, showing fatty infiltration. Again the treatment of other specimens with ether had no such effect, the cellular protoplasm having been replaced by a true fatty degeneration. The liver of yellow fever is harder and firmer than that of malarial fever, and is not readily acted upon by acids or alkalies. A piece of malarial liver placed in a mortar and treated with a solution of liquor potassæ is readily dissolved, the decoction having the appearance of venous blood, but no such action is produced by the same solution on the liver of yellow fever. A decoction of the liver may be made with either alcohol or water, the decoction of the malarial liver being of a yellowish brown, that of yellow fever of a golden yellow. This fatty infiltration of many of the tissues in yellow fever points to the fact that the albuminoid elements of the blood have undergone a retrograde metamorphosis, and that a proximate principle "fat," lower in the scale of organization, has been formed by the action of the yellow fever poison, whatever that may be.

The suprarenal capsules in hemorrhagic malarial fever are of a dark bronze color. Large deposits of pigment granules, but no fat.

In yellow fever the suprarenal capsules seem to have undergone fatty degeneration. Their tissue is also infiltrated with fat.

The kidneys in hemorrhagic malarial fever.—The exterior of the kidneys is of a dark bluish red, presenting here and there on their surface dark blackish spots; when opened, the same dark bluish red color is found. The whole organ is heavily congested; in fact, the lesion amounts to an active inflammation. All blood vessels, from the largest to the smallest, are distended with blood. There is evidence of hemorrhage in the malpighian corpuscles, filling a large number of the tubuli uriniferi with coagulated blood. These latter structures are in addition desquamated, and almost totally denuded of their pavement endothelium, and in many instances entirely so.

In hemorrhagic malarial fever the gall bladder is much distended, and filled with a viscid bile of a very dark green, almost black in appearance, and of a granular consistency.

The gall bladder in yellow fever is usually smaller than common, contracted and contains but little bile, and sometimes none.

In yellow fever the color of the kidneys is somewhat lighter than usual, owing to the presence of fat; at times we find a few dark spots beneath the capsule, but the principal changes are a fatty degeneration and a swelling of the uriniferous tubules, which seem, at times, almost denuded of their endothelium.

The bladder in hemorrhagic malarial fever is sometimes distended with highly colored urine, often containing blood corpuscles, large quantities of bile, epithelial cells, highly colored casts of tubuli uriniferi, frequently albumen, which is of a reddish brown color.

The bladder in yellow fever contains usually but little urine of a light yellow color, loaded with albumen of a golden yellow color, epithelial cells from the kidneys, casts of uriniferous tubules and urate of ammonia. Mucous membrane usually congested.

The brain in hemorrhagic malarial fever.--The dura mater nearly always presents a normal appearance, except that, in a few cases, I have found it slightly tinged with bile; the pia mater, on the other hand, was more deeply tinged with bile, its larger blood vessels were filled with blood, and the microscope disclosed the fact that the capillaries were also filled with blood. The gray substance of the cerebrum and cerebellum is usually of a blackish gray color, and its different layers difficult to distinguish. The dark ashy color of the cortical substance is due to an engorgement of the capillaries with blood of a dark granular appearance. The dirty yellowish color of the white substance is due to the same cause. This increased amount of blood in the brain renders its weight abnormally large. This congestion is ordinarily attended by a considerable effusion in the subarachnoid space, and not infrequently accompanied by an opacity of the arachnoid membrane. The anterior and posterior horns of the lateral ventricle, but especially the posterior horns, are in the majority of cases filled with an abnormal amount of yellowish serum; the larger vessels of the walls of the lateral and fourth ventricles, like the vessels of the pia mater give evidence of a heavy congestion. Urea is found in considerable quantity throughout the substance of the brain.

The brain in yellow fever.—Although some observers have asserted that inflammation of the brain exists in nearly all fatal cases of yellow fever, I must confess that I have not found it so. As a rule, I have found no very characteristic lesions. It is true, the microscope demonstrates a hyperæmic condition of the blood vessels of the brain, and it has been stated that a fatty degeneration of the blood vessels of the cortical substance and pia mater exists. An effusion of a yellow colored serum was found in the ventricles, which upon being tested showed the absence of bile. As in hemorrhagic malarial fever, urea is found in large quantity throughout the substance of the brain. In hemorrhagic malarial fever, all the ganglia of the sympathetic chain are of a yellow tinge, due to bile.

The sympathetic chain in yellow fever.—In the summer of 1873, I called the attention of the late Prof. H. D. Schmidt, of New Orleans, to the condition of the ganglia of the sympathetic chain. I found them of a deep pink color, and the microscope revealed an intensely congested condition of the blood vessels. This condition of these ganglia was noticed in an article of mine in the May number of the *New Orleans Medical Journal*, 1884. Subsequently Prof. Schmidt fully corroborated my statement, through his researches during the epidemic of 1878.

