# Correction

**Published:** 1898-07

**Authors:** 


**In Dr. Bat Smith's** article on the pathological anatomy of yellow fever etc., in our June issue, a typograhical error occurs which is of some importance. It is as follows: (See page 616.)

"This condition of these ganglia was noticed in an article of mine in the May number of the *New Orleans Medical Journal*, 1884."

The date should have been 1874; it is so written in the manuscript. We make the correction with pleasure. It establishes the doctor's claim to priority in the discovery of conditions mentioned; whereas Surgeon Gen. Sternburg accords it to Prof. H. D. Schmidt, Dr. Smith's preceptor.

